# Adeno-associated virus serotype-9 efficiently transduces the retinal outer plexiform layer

**Published:** 2009-07-17

**Authors:** Bo Lei, Keqing Zhang, Yongping Yue, Arkasubhra Ghosh, Dongsheng Duan

**Affiliations:** 1 Department of Ophthalmology, the First Affiliated Hospital of Chongqing Medical University, Chongqing Key Laboratory of Ophthalmology, Chongqing, China; 2 Department of Veterinary Medicine and Surgery, Department of Ophthalmology, Mason Eye Institute, University of Missouri, Columbia, MO; 3 Department of Molecular Microbiology and Immunology, University of Missouri, Columbia, MO

## Abstract

**Purpose:**

Adeno-associated virus serotype-9 (AAV-9) is a promising gene delivery vector. In this study, we evaluated AAV-9 transduction in the mouse retina.

**Methods:**

Three different AAV vectors were used in our study: AAV-9.RSV.AP, AAV-9.CMV.eGFP, and AAV-9.CMV.∆R4–23/∆C. In these vectors, two different promoters (the cytomegalovirus promoter-CMV promoter and the Rous sarcoma virus-RSV promoter) were used to express three different transgenes including the alkaline phosphatase (*AP*) gene, the enhanced green fluorescent protein (*eGFP*) gene, and a therapeutic microdystrophin gene (the *∆R4–23/∆C*). Specifically, 1 µl AAV-9 reporter gene vectors (1×10^9^ viral genome particles of AAV-9.RSV.AP or 1×10^10^ viral genome particles of AAV-9.CMV.eGFP) were administered subretinally to young (2–3-week-old), adult (3-month-old), and old (12-month-old) *C57BL/6J* mice. To evaluate AAV-9 transduction in a diseased retina, we injected subretinally 1×10^9^ viral genome particles of AAV-9.CMV.∆R4–23/∆C to *mdx^3cv^* mice, which we used as a model for Duchenne muscular dystrophy (DMD). Transgene expression was examined by histochemical as well as immunofluorescence staining at three and five weeks after injection. Electroretinograms were recorded five weeks after subretinal AAV-9.RSV.AP injection.

**Results:**

Subretinal injection yielded widespread transduction throughout the retina in all age groups. Robust expression was seen in the retinal pigment epithelium, outer nuclear layer, and in Müller cells. Interestingly a synaptic layer, the outer plexiform layer (OPL), also showed intensive expression. Transduction of the synaptic layer was further confirmed by immunostaining for C-terminal binding protein 2 (CtBP2), a marker for the photoreceptor synaptic ribbon. Dystrophin is normally expressed in the OPL photoreceptor terminals. This expression is lost in DMD patients and *mdx^3cv^* mice. Consistent with our findings in normal mice, we observed efficient microdystrophin expression in the OPL after AAV-9.CMV.∆R4–23/∆C infection. At five weeks after subretinal delivery of AAV-9.RSV.AP, no morphology or ERG abnormalities were observed.

**Conclusions:**

We demonstrated that AAV-9 is a potent vector for retinal gene delivery. Furthermore, subretinal AAV-9 administration did not cause appreciable acute retinal damages. In summary, AAV-9-mediated OPL transduction holds promise for treating diseases that primarily affect this layer.

## Introduction

Adeno-associated virus (AAV) is a single-stranded DNA virus. AAV-mediated gene therapy has successfully corrected several degenerative retinal diseases in animal models [1-4]. Recent successes in Leber congenital amaurosis phase I trials have provided the first clinical proof that AAV vector holds great promise in treating retinal diseases [5-8].

Recombinant AAV vectors are generated by replacing the endogenous viral genome with a therapeutic or marker gene expression cassette. The prototype AAV vector is based on AAV serotype-2 (AAV-2). In the last few years, several new AAV serotypes have been developed [9]. Due to the differences in the cellular transduction pathway, these new serotypes have opened additional avenues for tailoring AAV gene therapy to specific clinical applications. Previous studies have evaluated AAV serotypes 1–9 in the retina [3,10-13]. It was found that subretinal injection transduces the outermost retinal structures, such as retinal pigment epithelium (RPE) and the photoreceptors, while intravitreal injection transduces ganglion cells in the innermost layer. Pathology in the retinal synaptic layers such as the outer plexiform layer (OPL) is associated with a wide range of retinal diseases [14-18]. Two recent studies suggest that AAV may transduce the OPL [12,19]. However, these studies did not explicitly explore the specificity of OPL transduction. Considering the importance of the OPL in retinal diseases, a thorough and more focused study would be necessary to establish AAV transduction in the OPL. Such efforts would likely open the door for AAV-mediated OPL disease gene therapy.

AAV serotype-9 (AAV-9) was discovered a few years ago from human tissue [9,20]. Due to its unique serological property, it was classified as clade F, a clade distinctive from all known AAVs [20]. AAV-9 has been shown to efficiently transduce several tissues including the heart, liver, lung, kidney, pancreas, and skeletal muscle [21-27]. Furthermore, it was reported recently that AAV-9 is capable of bypassing the blood-brain barrier and efficiently targeting cells of the central nervous system [21]. This unique feature may enable the development of gene therapies for a range of neurodegenerative diseases. Two studies evaluated AAV-9 transduction in the retina following subretinal administration [12,13]. Both studies demonstrated efficient transduction of RPE and Müller cells [12,13]. Interestingly, one group showed photoreceptor transduction [12]. However, the other group did not detect photoreceptor transduction [13]. The reasons for these differences are not clear but may relate to animal age, the promoter and the reporter gene used, and the time frame of observation. To further characterize AAV-9 transduction in the retina, we performed a comprehensive study in young (2–3-week-old), adult (3-month-old), and old (12-month-old) mice using either an Rous sarcoma virus promoter (RSV) driving alkaline phosphatase reporter gene vector (AAV-9.RSV.alkaline phosphate [AP]) or a Cytomegalovirus promoter (CMV) driving enhanced green fluorescent protein (AAV-9.CMV.enhanced green fluorescent protein [eGFP]) reporter gene vector. To further extend the study, we also evaluated subretinal delivery of a therapeutic microdystrophin vector (AAV-9.CMV.∆R4–23/∆C) in adult *mdx^3cv^* mice, a model for Duchenne muscular dystrophy (DMD). We observed widespread transduction throughout the retina following subretinal injection. Interestingly, we observed remarkable transduction in the synaptic OPL irrespective of the vector used. Colocalization experiments with a marker of the photoreceptor presynaptic ribbon further confirmed the OPL transduction. To our knowledge, this is the first demonstration of efficient retinal synaptic layer transduction by an AAV vector. Besides tissue tropism, we also evaluated acute toxicity of subretinal AAV-9 injection. Our results suggest that subretinal AAV-9 delivery was not associated with deleterious effect on retinal morphology and electroretinogram (ERG) function.

## Methods

### Experimental animals

All experiments were conducted in accordance with the Association for Research in Vision and Ophthalmology Statement for the Use of Animals in Ophthalmic and Vision Research. The experimental protocols were reviewed and approved by institutional animal care and use committee at the University of Missouri. *C57BL/6J* and *mdx^3cv^* mice were originally purchased from The Jackson Laboratory (Bar Harbor, ME). Experimental mice were obtained from local breeding colonies. All mice were housed in specific pathogen-free (SPF) animal care facilities and kept under a 12 h:12 h light-dark cycle (25 lx) with free access to food and water.

### Recombinant *AAV-9* production

AAV-9 stocks were generated using an adenoviral free triplasmid transfection protocol described previously [22,23]. Briefly, 70% to 80% confluent 293 cells were cotransfected with a *cis*-plasmid, a pRep2/Cap9 helper plasmid (a gift from Dr. James M. Wilson, University of Pennsylvania, Philadelphia, PA) and an adenoviral helper plasmid (pHelper; Stratagene, La Jolla, CA). Crude viral lysate was harvested 72 h later and purified through two rounds of CsCl isopycnic ultracentrifugation. Purified virus was dialyzed through three exchanges of HEPES buffered saline at 4 °C. Viral titer determination and quality control were performed as described before [22,23]. Briefly, viral DNA was extracted and slot blot hybridization was performed using P32-labeled radioactive probe. Vector genome was determined according to the plasmid copy number standards. The absence of significant wild type AAV contamination was confirmed by immunofluorescence staining with an antibody that recognizes AAV Rep protein.

The *cis*-plasmids (pcisRSV.AP, pcisCMV.eGFP, and pcisCMV.∆R4–23/∆C) have been reported previously [24,25]. pcisRSV.AP expresses the heat-resistant human placental AP gene under the transcriptional control of the RSV promoter and the SV40 polyadenylation signal (pA). pcisCMV.eGFP expresses the eGFP gene under the transcriptional control of the CMV promoter and the SV40 pA. pcisCMV.∆R4–23/∆C encodes a highly abbreviated ∆R4–23/∆C human microdystrophin gene under the transcriptional control of the CMV and the SV40 pA [28]. Specifically, a large portion of the dystrophin rod domain (from spectrin-like repeat 4 to 23) and the entire C-terminal domain are deleted. The recombinant AAV vectors are called AAV-9.RSV.AP, AAV-9.CMV.eGFP, and AAV-9.CMV.∆R4–23/∆C.

### Subretinal injection

AAV-9.RSV.AP and AAV-9.CMV.eGFP injection were delivered to young (2- to 3-week-old), adult (3-month-old), and old (12-month-old) *C57BL/6J* mice. AAV-9.CMV.∆R4–23/∆C injection was delivered to 3-month-old adult *mdx^3cv^* mice. HEPES-buffered saline was used as vehicle control in a subset of animals.

Mice were given 1% atropine eyedrops 3 h before they were anesthetized with an intraperitoneal injection of a mixture of 75 mg/kg ketamine and 13.6 mg/kg xylazine. Following general anesthesia, 2.5% phenylephrine hydrochloride eyedrops were applied. One drop of 1% proparacaine hydrochloride was administered as local anesthesia, followed by 2.5% hydroxypropyl methylcellulose. Subretinal injection was performed according to a previous publication with modifications [29]. Briefly, an aperture within the pupil area was made through the superior cornea with a 30-gauge needle. A 33-gauge blunt needle mounted on a 10-μl syringe was introduced through the corneal opening, avoiding the lens and penetrating the neuroretina to reach the posterior subretinal space. The NanoFil™ sub-microliter injection system (WPI, Sarasota, FL) was used to inject 1 µl of AAV vector in 30 s. The injection was considered successful when retinal blebs occupied more than half of the retina. Evaluation was performed only in mice that were successfully injected. Following subretinal injection, 1% atropine eyedrops and antibiotic ophthalmic ointment were administered daily for three days. Seven days after injection, the mice were anesthetized with ketamine and xylazine as described previously, and their eyes were examined under microscope. Eyes that exhibited any sign of surgical complications, including anterior or posterior synechia, cataract, vitreous and retinal hemorrhage, and unresolved retinal detachment, were excluded from the study. Such signs were observed in 20% to 30% of the eyes.

### Retinal morphology and gene expression

For AP and dystrophin expression, mouse eyes were enucleated and snap frozen in liquid nitrogen–cooled isopentane in optimal cutting temperature (OCT) compound (Sakura Finetek Inc., Torrance, CA). Next, 10 µm cryosections were cut along the optic nerve head and stained with hematoxylin and eosin (H&E) for routine retinal morphology. After endogenous heat labile AP activity was heat inactivated, AAV-9 mediated AP expression was examined by histochemical staining using a previously described protocol [22-24]. Microdystrophin expression was evaluated by immunofluorescence staining according to a previously published protocol [30]. Briefly, cryosections were sequentially blocked with Papain-digested rabbit anti-mouse immunoglobulin and rabbit serum. Then sections were stained with the primary anti-dystrophin antibody and signal was detected with appropriate secondary antibodies. The present study employed 1:30 Dys-2 antibody (Novocastra, Newcastle, UK), which recognizes dystrophin C-terminal domain and 1:20 Dys-3 antibody (Novocastra), which recognizes the hinge 1 region in human dystrophin. Also used was 1:100 Alexa Fluor 594-conjugated goat anti-mouse IgG (H^+^L) F(ab’)_2_ fragment antibody as the secondary antibody in immunofluorescence staining (Invitrogen, Carlsbad, CA). For eGFP and CtBP2 detection, the eyeballs were first fixed in 4% paraformaldehyde and phosphate-buffer saline (PBS,, 0.01 M phosphate buffer, 0.0027 M potassium chloride and 0.137 M sodium chloride, pH 7.4) then cryoprotected in 30% sucrose/PBS overnight. Next, 10 µm cryosections were stained with 1:200 goat anti-CtBP2 (C-terminal binding protein 2, Santa Cruz, Biotechnology, Santa Cruz, CA) followed by 1:500 Texas Red conjugated donkey anti-goat IgG secondary antibody (Jackson ImmunoResearch, West Grove, PA). Nuclei were revealed with 1:500 4’,6-diamidino-2-phenylindole, dihydrocholoride (DAPI, Molecular Probes, Eugene, OR). The specificity of immunostaining was confirmed by staining cryosections in the absence of the primary antibody. Photomicrographs were taken with a digital camera using a Leica fluorescence microscope (DMR, Deerfield, IL).

### Electroretinography

Retinal function was evaluated at 5 weeks after subretinal injection. Both dark- and light-adapted ERG were examined according to a previously published protocol [31,32].

### Statistical analysis

Data are presented as mean±standard deviation (mean±SD). Statistical significance was examined with ANOVA followed by the Tukey post hoc test. A p value of less than 0.05 was considered as significant.

## Results

**Figure 1:**
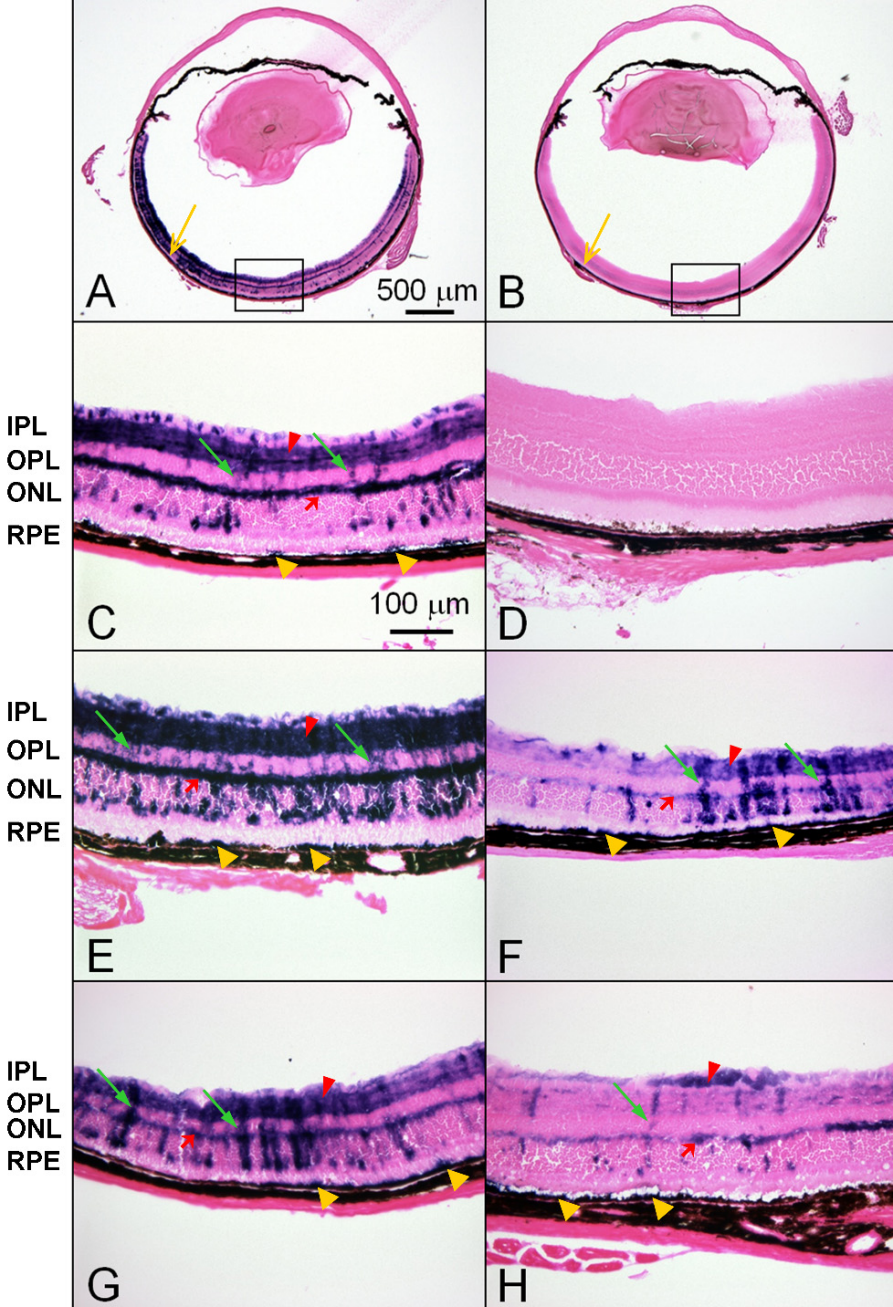
Representative retinal cross sections of *C57BL/6J* mice after subretinal delivery of AAV-9.RSV.AP. Panels **A** and **B** show global view of AAV-9 transduction in adult (3-month-old) *C57BL/6J* mouse eyes at 5 weeks after subretinal AAV-9.RSV.AP injection (**A**) and HEPES buffer injection (**B**). Yellow arrows indicate the injection sites. Panels **C** and **D** show the enlarged retinal sections of the boxed regions in **A** and **B**, respectively. Panels **E** and **F** are representative retinal sections obtained from young (3-week-old) mice at 3 weeks after subretinal injection. **E** shows the area close to the injection site while **F** shows an area distant from the injection site. Panels **G** and **H** are representative retinal sections obtained 5 weeks after subretinal injection in a 12-month-old mouse. **G** shows the area close to the injection site while **H** shows an area distant from the injection site. Similar AP expression pattern can be seen in all AAV-9.RSV.AP-injected eyes. Dark blue AP staining is readily visible throughout the entire retina. The RPE, ONL, INL, and RGC layers show punctate staining. AP-positive Müller cells display staining across majority of the retina thickness from the ONL to the RGC layers. AP expression in the OPL and the IPL layers is prominent. Although staining in the region distant from the injection site is weaker, AP expression is widespread in the two synaptic layers. Representative photomicrograph from a HEPES buffer mock-infected eye shows no evidence of AP expression. Yellow arrows mark the injection site; red arrows indicate outer plexiform layer (OPL); red arrowheads indicate inner plexiform layer (IPL); green arrows indicate Müller cells; yellow arrowheads indicate RPE. Abbreviations: alkaline phosphatase (AP), inner nuclear layer (INL), outer nuclear layer (ONL), retinal ganglion cell (RGC), retinal pigment epithelium (RPE).

We first examined subretinal AAV-9 AAV.RSV.AP injection in normal *C57BL/6J* mice. Figure 1 shows the representative retinal cross-sections from young (n=6 eyes, 3 weeks post-injection), adult (n=12 eyes, 5 weeks post-injection) and old (n=5 eyes, 5 weeks post-injection) mice. AAV-9 resulted in widespread (peripheral to central to peripheral) and throughout (RPE to retinal ganglion cells [RGC]) AP expression in all age groups (Figure 1A, data not shown for young and old mice). Nevertheless, the highest expression was observed at the injection site (Figure 1A, arrow). AP expression was observed in the RPE, outer nuclear layer (ONL), inner nuclear layer (INL), OPL, inner plexiform layer (IPL), RGC layer and Müller cells but not the outer and inner segments of the photoreceptor (Figure 1C,E-H). Remarkably, two retinal synaptic layers (OPL and IPL) were highly transduced (Figure 1C,E-H). AP expression was not detected in the mock-infected control eyes (5 eyes each for young and adult mice; Figure 1B,D).

**Figure 2:**
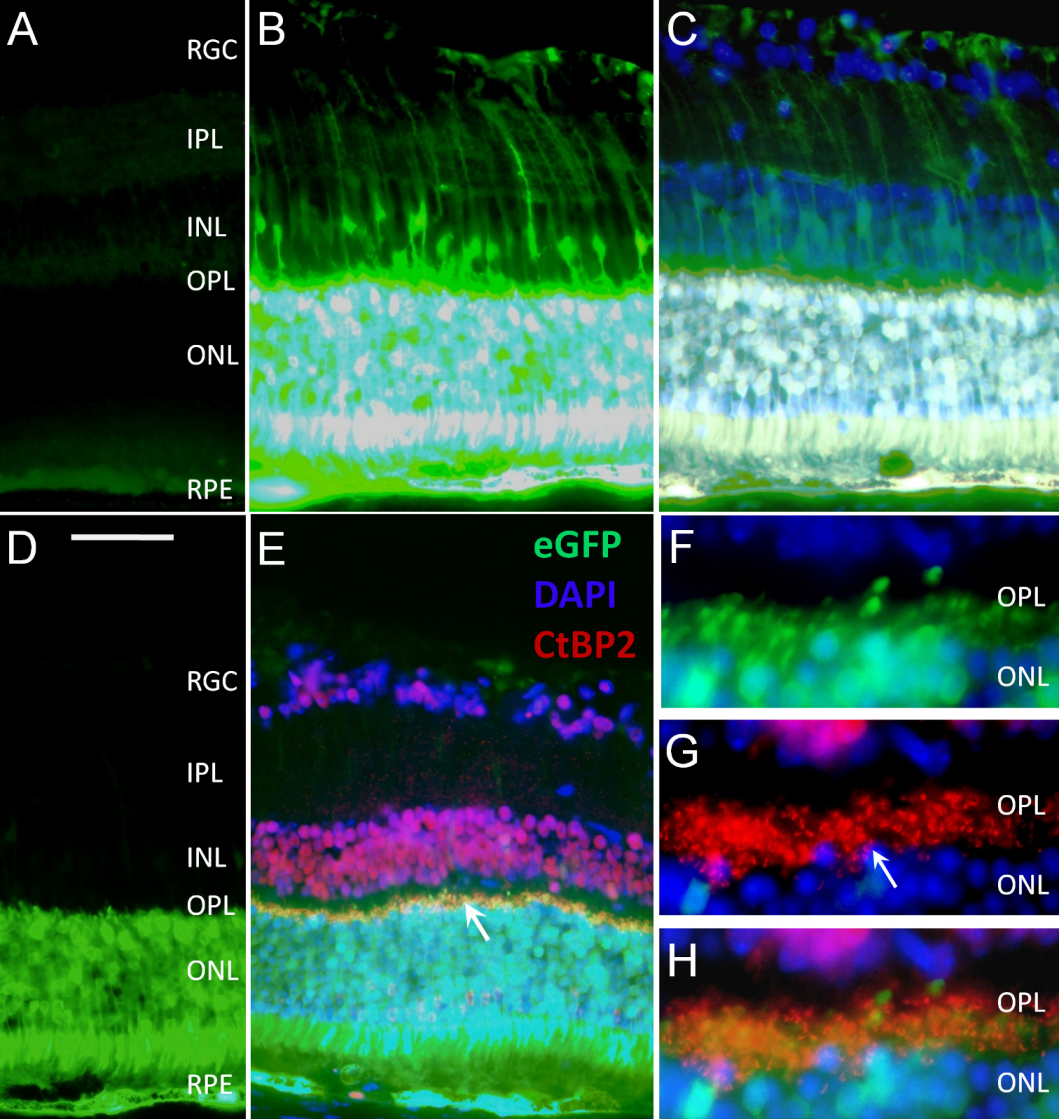
Retinal eGFP expression after subretinal delivery of an AAV-9.CMV.eGFP vector in a 3-month-old *C57BL/6J* mouse. AAV-9 led to widespread (from the injection site, which is close to the posterior pole, to the peripheral retina) and throughout (from the outer retina RPE layer to the inner retina RGC layer) eGFP expression in the mouse retina. The eGFP expression pattern near the injection site and in areas remote from the injection site was similar. The pictures were taken at approximately 300 to 500 µm away from the injection site. **A**-**C** were taken under the same exposure conditions. **D**-**H** were taken with a shorter exposure time. **A** is a section from a control eye. **B** shows eGFP expression in the retina, and **C** is a merged picture of **B** and DAPI staining. **B** and **C** show GFP expression in the RPE, photoreceptors (including the outer and inner segment), ONL, OPL, Müller cells in the INL, IPL and RGC layer. Because of a shorter exposure time (**D**), eGFP expression was only seen in the outer retina including RPE and the photoreceptor layer. No expression was observed in the inner retina. **E** is a merged picture of eGFP expression and CtBP2, DAPI staining. In the distal portion of the OPL, colocalization of eGFP expression and CtBP2 staining is evident (arrow). **F, G**, and **H** are enlarged pictures of the OPL. **F**. eGFP expression is evident in the distal portion of the OPL, which is beyond the photoreceptor nuclei (in blue). **G** shows CtBP2 staining (arrow) in the photoreceptor terminals. Panel **H** is a merged picture of eGFP expression and CtBP2, DAPI staining. eGFP expression overlaps with CtBP2 in the photoreceptor terminals in the distal portion of the OPL The calibration bar is 50 μm for **A**-**E**, and 20 μm for **F**-**H**. Abbreviations: inner nuclear layer (INL), inner plexiform layer (IPL), outer nuclear layer (ONL), outer plexiform layer (OPL), retinal ganglion cell (RGC), retinal pigment epithelium (RPE).

To exclude the potential bias from the transgene as well as the promoter, we evaluated AAV-9.CMV.eGFP transduction (4 eyes in 2-week-old and 4 eyes in 3-month-old mice). Four weeks after subretinal injection, we observed intense eGFP expression in young and adult groups in the RPE, ONL, INL, OPL, IPL, RGC layers, and Müller cells (Figure 2B,C). Interestingly, we also observed robust expression in the photoreceptors (Figure 2B-E). To further confirm OPL transduction, we stained AAV-9.CMV.eGFP-infected eyes with CtBP2, a marker for the photoreceptor synaptic ribbon (Figure 2 E,G,H). The colocalization of eGFP and CtBP2 strongly suggest that the OPL layer was transduced (Figure 2F,H).

OPL defects are associated with several retinal diseases. To determine whether AAV-9 can be used to deliver a therapeutic gene to the OPL, we performed subretinal injection in *mdx^3cv^* mice using AAV-9.CMV.∆R4–23/∆C vector. *Mdx^3cv^* mice are models for DMD, a lethal childhood genetic disease caused by mutations in the dystrophin gene [33,34]. Besides muscle disease, DMD patients also suffer from pathology in other systems such the central nerve system. A 260 kDa dystrophin isoform (Dp260) is normally expressed in the OPL. In the eyes of DMD patients and *mdx^3cv^* mice, Dp260 expression is lost [35-37]. The absence of Dp260 has been associated with the abnormal ERG seen in DMD patients.

**Figure 3:**
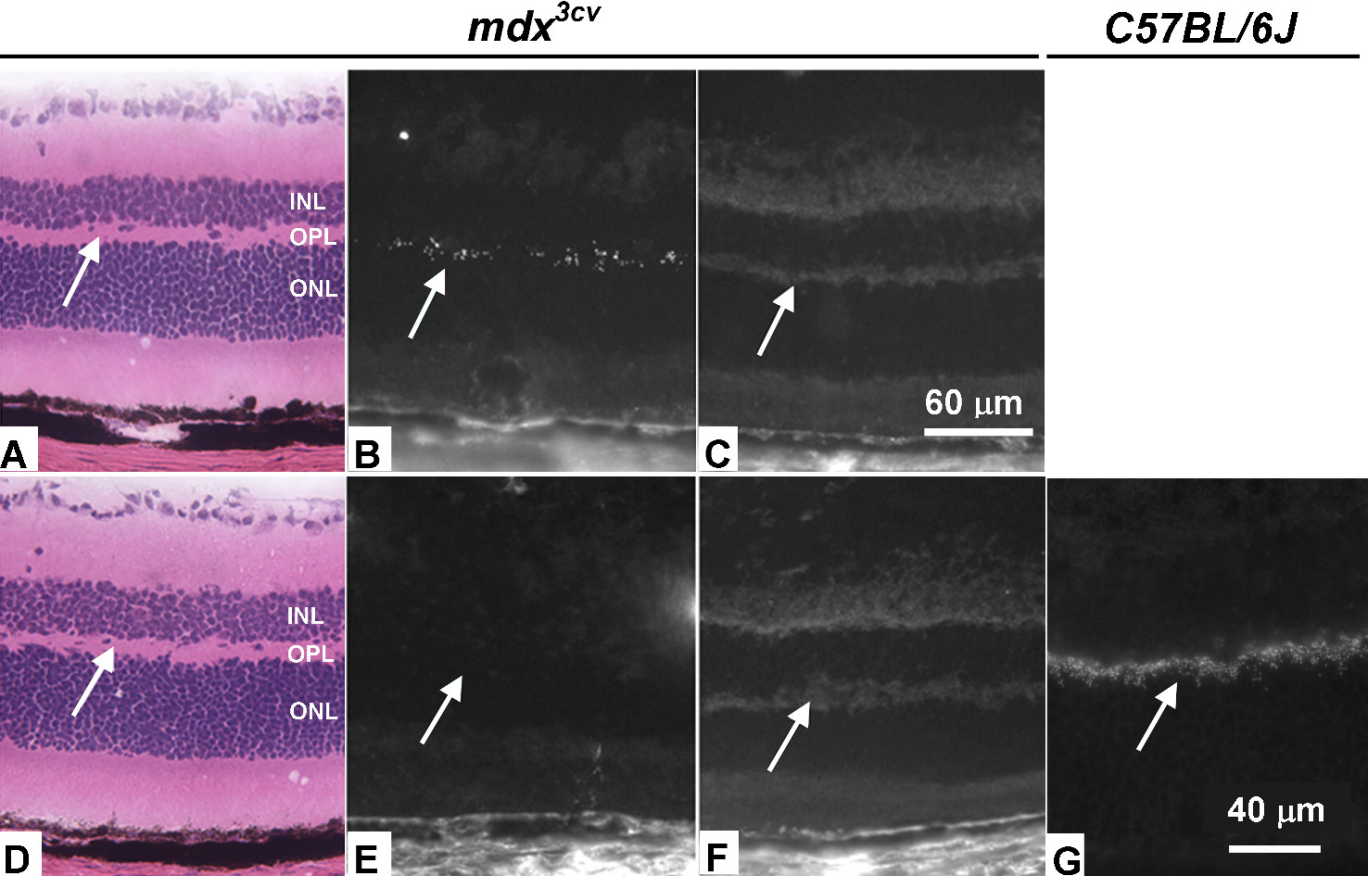
Retinal microdystrophin expression in gene transferred 3-month-old *mdx^3cv^* mouse. Subretinal delivery of an AAV-9 human microdystrophin vector resulted in efficient OPL transduction in *mdx^3cv^* mouse retina. **A**-**F** are from *mdx^3cv^* mouse eyes, and **G** is from a *C57BL/6J* mouse eye. **A**-**C** are representative serial sections from an eye infected with AAV-9 microdystrophin vector. **D**-**F** are representative serial sections from an eye mock-infected with HEPES buffer. **A, D** show retinal structure of *mdx^3cv^* mouse (H&E staining). **B, E** show immunostaining with the Dys-3 antibody, which recognizes microdystrophin. At 5 weeks after subretinal injection, microdystrophin expression was evident in the injected retina (**B**), but not in the mock-infected eye (**E**). **C** and **F** display immunostaining with the Dys-2 antibody, which recognizes endogenous dystrophin. Neither the AAV-9-infected nor the mock-infected eye showed endogenous dystrophin expression. **G** shows immunostaining with the Dys-2 antibody on the *C57BL/6J* retina. Dystrophin expression is seen in the outer plexiform layer (OPL). Arrows point to the OPL. Abbreviations: inner nuclear layer (INL), outer nuclear layer (ONL).

The 3.8 kb ∆R4–23/∆C microgene encodes a highly truncated dystrophin. This microgene has been extensively studied as a candidate gene for DMD gene therapy [28]. At 5 weeks after subretinal injection (n=5 mice; AAV-9.CMV.∆R4–23/∆C to one eye and HEPES buffer to the contralateral eye), we evaluated dystrophin expression by immunofluorescence staining. Two epitope-specific antibodies were used in the study. The Dys-2 antibody recognizes endogenous Dp260 in the wild type retina (Figure 3G) [38], while the Dys-3 antibody only reacts with microdystrophin. Consistent with our findings in reporter AAV vector-infected normal eyes, we observed efficient OPL transduction in the eyes of AAV-9.CMV.∆R4–23/∆C infected *mdx^3cv^* mice (Figure 3B,C). No dystrophin was detected in HEPES buffer-injected eyes (Figure 3E,F).

**Figure 4:**
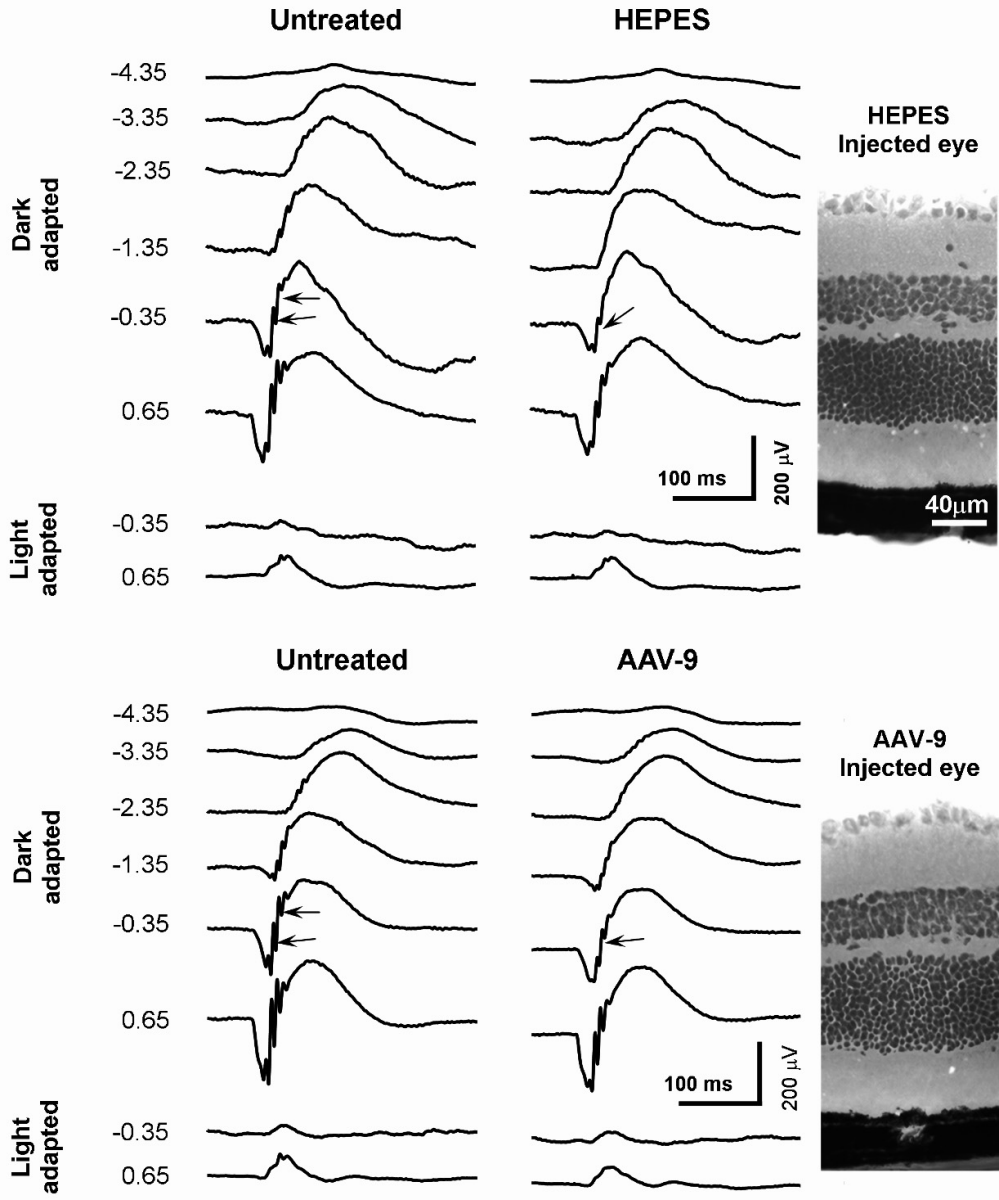
Effects of subretinal delivery of an AAV-9.RSV.AP vector on mouse electroretinogram. Dark-adapted and light-adapted electroretinograms were recorded from 12-month-old *C57BL/6J* mice at 5 weeks after subretinal injection of AAV-9.RSV.AP or HEPES buffer. One eye was injected and the contralateral eye was untreated. Black arrows indicate OPs. The numbers to the left of the ERG signals represent the stimulus light intensity (log cd-s/m^2^). The background light for light-adaptation was 30 cd/m^2^.

**Figure 5:**
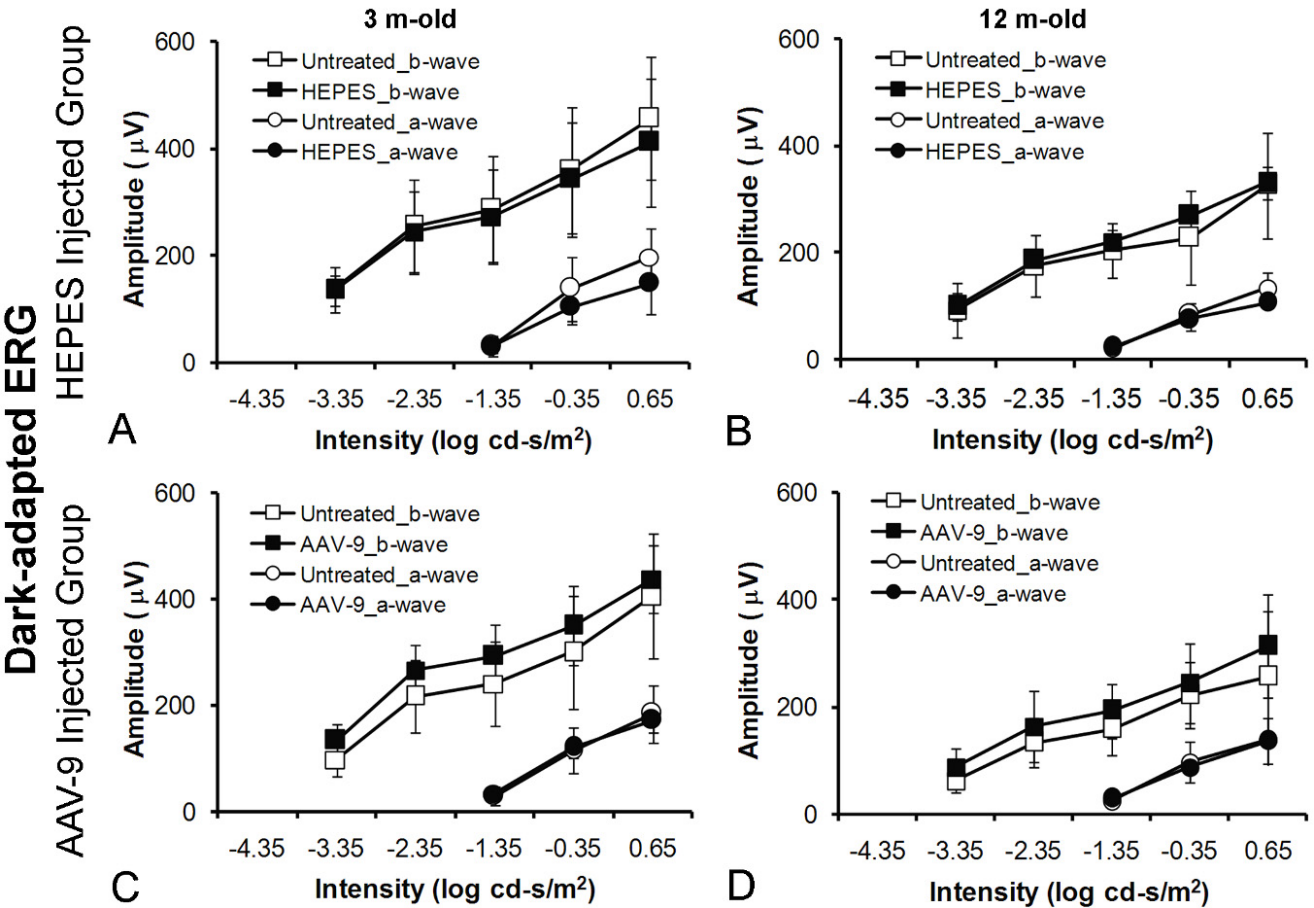
Dark-adapted ERG a-wave and b-wave responses-intensity curves in *C57BL/6J* mice. In all animals, one eye was injected subretinally while the contralateral eye was untreated. **A, B** show HEPES-treated mice and **C, D** show AAV-9.RSV.AP-injected mice. The left column shows the 3-month-old groups (n=5 for each group) and the right column shows the 12-month-old groups (n=5 for each group). The filled symbols represent the injected eyes, and the open symbols represent the untreated eyes. Error bars indicate the standard deviation from the mean (mean±SD).

**Figure 6:**
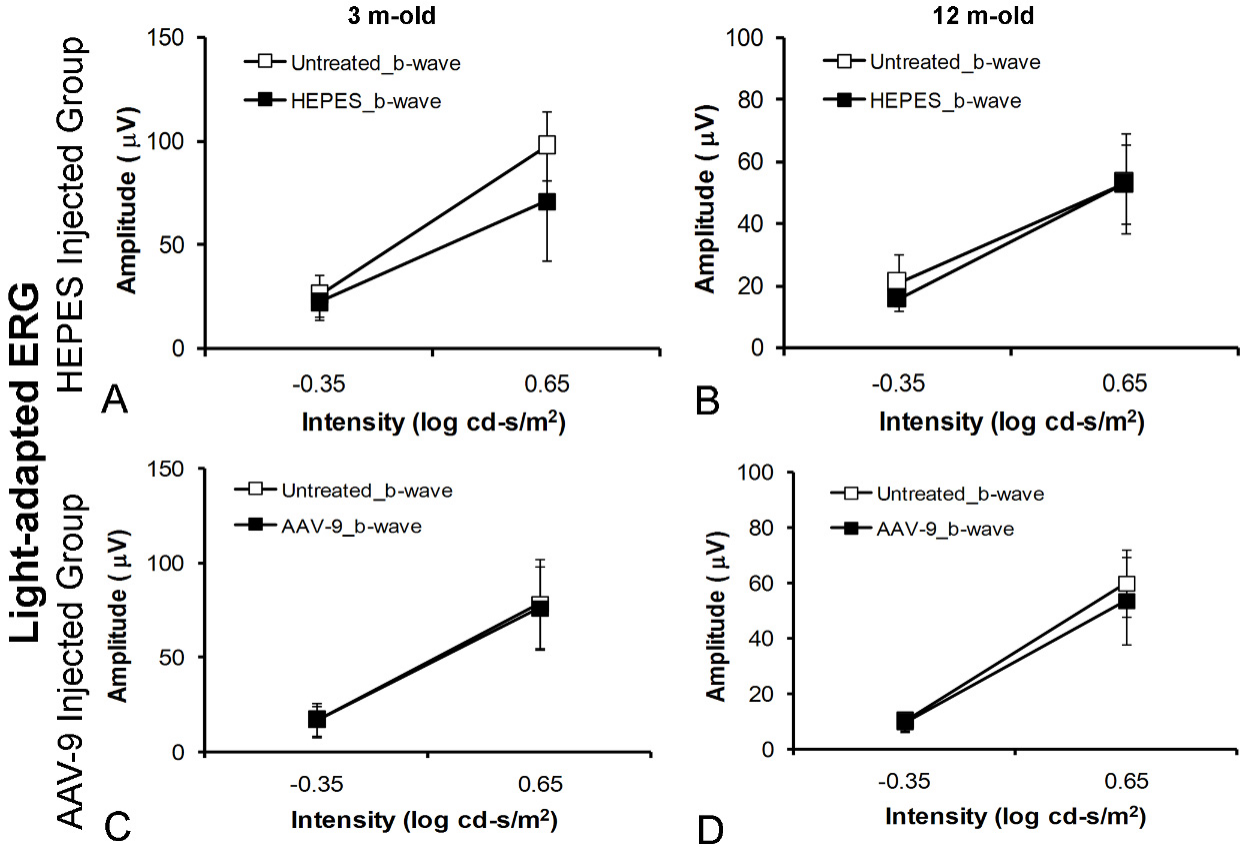
Light-adapted ERG b-wave responses-intensity curves in *C57BL/6J* mice. In all the animals, one eye was injected subretinally while the contralateral eye was not treated. **A, B** show HEPES-treated mice and **C, D** show AAV-9.RSV.AP-injected mice. The left column shows the 3-month-old groups (n=5 for each group), and the right column shows the 12-month-old groups (n=5 for each group). The filled symbols represent the injected eyes, and the open symbols represent the untreated eyes. Background light was 30 cd/m^2^. Error bars indicate the standard deviation from the mean (mean±SD).

Next, we studied whether subretinal AAV-9 injection causes acute retinal damage. At 5 weeks after subretinal injection (AAV-9.RSV.AP or HEPES) in adult and old C57BL/6J mice (n=5 for each age group), we examined retinal histology and recorded dark-adapted and light-adapted ERGs. Compared with untreated eyes, neither HEPES buffer injection nor AAV-9.RSV.AP injection resulted in appreciable morphology alterations (Figure 4). In each age group, the thresholds and amplitudes of the dark-adapted ERG a-wave, dark-adapted b-wave and light-adapted b-wave of the AAV-9 or HEPES injected eyes were comparable to those of the untreated eyes (p>0.05, Figure 4, Figure 5, and Figure 6). On the ascending limb of dark-adapted b-wave, the amplitude of the oscillatory potentials (OPs) in the AAV-9 vector injected eyes appeared smaller than the untreated eyes (Figure 4). However, similar reduction was also observed in HEPES-injected eyes.

## Discussion

AAV-mediated gene therapy holds great promise for rescuing vision loss in retinal degenerative diseases. Remarkable progress has been made in animal models and human patients [1,2,4,6-8]. However, most studies have focused on diseases that primarily affect the photoreceptor, RPE, and RGC layer.

There are two synaptic layers in the retina including the OPL in the outer retina and the IPL in the inner retina. The OPL and IPL are indispensable for mediating visual signal transmission. While no human diseases have been associated with the IPL, several retinal diseases originate in as well as primarily affect the OPL. Examples of these diseases include congenital stationary night blindness, retinoschisis, DMD retinopathy, and melanoma-associated retinopathy [14-18]. Gene therapy for these diseases is largely dependent on a vector that can efficiently deliver the therapeutic genes to the OPL.

Several different AAV serotypes have been evaluated for retinal gene delivery. In this study we examined AAV-9 transduction in young, adult, and old mice. Consistent with recent publications from other investigators, we observed robust AAV-9 transduction in the outer retina (RPE and ONL), inner retina (Müller cells), and RGC layers [12,13]. Surprisingly, we also observed quite efficient and widespread transduction in the synaptic layers (Figure 1, Figure 2, and Figure 3). A colocalization study suggested that transduction occurred in the photoreceptor terminals, but not in the bipolar cell and horizontal cell dendrites in the OPL (Figure 2).

To explore therapeutic gene delivery to the OPL we delivered AAV-9.CMV.∆R4–23/∆C to adult *mdx^3cv^* mice. We focused on transgene expression rather than functional correction in this study. Consistent with our findings in normal mice, we observed robust microdystrophin expression in the OPL. Taken together, our results suggest that AAV-9-mediated OPL transduction represents a promising approach to treat retinal diseases that are related to OPL defects.

It is important to understand whether subretinal administration of a new viral vector causes any side effects. We found that subretinal delivery of AAV-9 did not alter retinal structure. In addition, rod and cone photoreceptor and bipolar cell functions were not affected by subretinal AAV-9 injection (Figure 4, Figure 5, and Figure 6). Interestingly the OPs, highly sensitive functional indicators of the inner retina [39], were reduced following AAV-9 injection. Since similar reductions were also observed in HEPES-buffer-injected eyes. We suspect that the OP reduction may relate to the injection procedure rather than AAV-9 vector.

Taken together, our data suggest that AAV-9 is a potent vector for retina gene delivery, and subretinal administration does not cause acute damage. Efficient transduction of the photoreceptor terminals opens the door to develop AAV-9 gene therapy for degenerative retinal diseases that affect this synaptic structure.
